# Validation of the prognostic Heidelberg re-irradiation score in an independent mono-institutional patient cohort

**DOI:** 10.1186/1748-717X-9-128

**Published:** 2014-06-03

**Authors:** Maximilian Niyazi, Maya Flieger, Ute Ganswindt, Stephanie E Combs, Claus Belka

**Affiliations:** 1 Department of Radiation Oncology, University of Munich, Marchioninistr. 15, 81377 Munich, Germany; 2 Department of Radiation Oncology, University of Heidelberg, Im Neuenheimer Feld 672, 69120 Heidelberg, Germany; 3 Department of Radiation Oncology, Klinikum rechts der Isar der TU München, Ismaninger Str. 22, 81675 Munich, Germany

**Keywords:** Bevacizumab, Re-irradiation, Radiotherapy, Glioma, Glioblastoma, Heidelberg score, Prognostic

## Abstract

**Purpose:**

Re-irradiation has been shown to be a valid option with proven efficacy for recurrent high-grade glioma patients. Overall, up to now it is unclear which patients might be optimal candidates for a second course of irradiation. A recently reported prognostic score developed by Combs et al. may guide treatment decisions and thus, our mono-institutional cohort served as validation set to test its relevance for clinical practice.

**Patients and methods:**

The prognostic score is built upon histology, age (< 50 vs. ≥ 50 years) and the time between initial radiotherapy and re-irradiation (≤ 12 vs. > 12 months). This score was initially introduced to distinguish patients with excellent (0 points), good (1 point), moderate (2 points) and poor (3–4 points) post-recurrence survival (PRS) after re-irradiation. Median prescribed radiation dose during re-treatment of recurrent malignant glioma was 36 Gy in 2 Gy single fractions. A substantial part of the patients was additionally treated with bevacizumab (10 mg/kg intravenously at d1 and d15 during re-irradiation).

**Results:**

88 patients (initially 61 WHO IV, 20 WHO III, 7 WHO II) re-irradiated in a single institution were retrospectively analyzed. Median follow-up was 30 months and median PRS of the entire patient cohort 7 months. Seventy-one patients (80.7%) received bevacizumab. PRS was significantly increased in patients receiving bevacizumab (8 vs. 6 months, p = 0.027, log-rank test). KPS, age, MGMT methylation status, sex, WHO grade and the Heidelberg score showed no statistically significant influence on neither PR-PFS nor PRS.

**Conclusion:**

In our cohort which was mainly treated with bevacizumab the usefulness of the Heidelberg score could not be confirmed probably due to treatment heterogeneity; it can be speculated that larger multicentric data collections are needed to derive a more reliable score.

## Introduction

In patients with high-grade glioma (HGG) a substantial rate of local failures has been observed after multimodal therapy [1]. The addition of temozolomide (TMZ) increased local control and survival, whereas the 2-year survival rate remained 27.2% [2].

In selected patients, a second course of radiotherapy (RT) was shown to be a reasonable treatment option [3-5]. One highly important question is which patients should be candidates for a second course of irradiation as not all patients seem to profit from such a second course. Concerning e. g. re-surgery, such a score was derived by Park and colleagues including KPS, tumor volume and the MSM score, which could be validated in an independent patient dataset and was therefore even predictive for patients undergoing re-surgery [6]. Thus, Combs and colleagues developed a prognostic score in order to estimate the survival benefit of patients who are planned to be irradiated [7], whereas no validation was performed by this group.

Therefore, we aimed at a validation in our independent patient cohort. One major difference between the initial and our cohort was the additional application of bevacizumab in a substantial part of the cases.

Various groups have already investigated the use of bevacizumab – a humanised monoclonal antibody against VEGF-A with an already established role in metastatic colon, breast, and lung cancer [8] – for patients with recurrent HGG [9] and several trials have documented its efficacy [10-14], which may be due to the presence of pronounced hypoxia as well as high levels of tumor driven angiogenesis in HGG [15,16].

Since the efficacy of radiation-based re-treatment is limited, it is reasonable to test in how far the addition of a radiation response modulator would impact on the efficacy of re-treatment. In this regard, Gutin and co-workers determined the safety and activity of RT and concomitant bevacizumab – for the GBM cohort, PFS-6 was 65% [17]. In a previous retrospective study on 30 patients, 20 being treated with bevacizumab we could show that PFS-6 within the bevacizumab-treated cohort was 72% and survival was significantly enhanced [18]. After the publication of latter initial results we extended the use in clinical practice. Thus, the value of this approach was determined retrospectively by comparing the outcomes of patients having received a bevacizumab based re-irradiation treatment with those being re-treated without bevacizumab with a higher case number and substantially longer follow-up [19]. The advantage of adding bevacizumab was still present in this updated analysis.

The aim of this study is to present the results after retrospective determination of the Heidelberg score compared to outcome data and to test its prognostic significance.

## Patients and methods

### Patient selection

Only patients with histologically and/or FET-PET/MRI proven recurrence and macroscopic tumor (maximum diameter 5 cm with few exceptions, multifocality per se was no contraindication) were admitted to re-irradiation, the interval between first radiotherapy and re-irradiation had to be 6 months at minimum. Patients that received alternative treatment modalities, e. g. complete resection by re-surgery, interstitial brachytherapy or systemic chemotherapy were excluded from the analysis.

### Treatment schedule and follow-up

Before treatment, a gadolinium-enhanced brain MRI with gradient echo sequence and perfusion and/or a [^18^ F]FET-PET were performed. Patients treated with bevacizumab received 10 mg/kg at days 1 and 15 during radiotherapy. If applied in patients who had no previous progression after TMZ pre-treatment a dosage of 75 mg/m^2^ daily was chosen.

Treatment outcome was evaluated on a regular basis (every three months) by brain MRI [20] and/or FET-PET. Adjuvant chemotherapy was prescribed on an individual base as no standard has been defined yet but was not set as mandatory.

### Radiotherapy

By analogy with Combs et al. [21] patients received a total dose of 36 Gy in 18 fractions (2 Gy single doses) employing 3D conformal radiotherapy or IMRT if adjacent critical structures were present. Planning target volume (PTV) was defined as gross tumor volume (GTV) plus 10 mm margin at maximum. GTV included the contrast enhancing lesion in T1w + Gd MRI. To ensure reproducibility patients were immobilized with a thermoplastic mask system. Treatment planning was performed using the Oncentra® treatment planning system (OTP MasterPlan®, Nucletron, Solingen, Germany).

### Toxicity evaluation

Adverse events and toxicity were determined retrospectively using the National Cancer Institute’s Common Toxicity Criteria, version 4.0 as reported before [18,22]. Concerning adverse events of radiotherapy, focus was set on radiation necrosis as well as generalized leukoencephalopathy.

### Statistics

Outcome measures of this retrospective analysis were overall survival for the entire cohort from initial treatment, safety of bevacizumab given in combination with RT for recurrent HGG as well as post-recurrence and progression-free survival (PRS & PR-PFS) in patients treated with or without bevacizumab. Survival analyses were based on Kaplan-Meier estimates, univariate modelling was based on the logrank-test. For all patients, PRS was measured from the first day of re-irradiation until death or last follow-up and progression-free survival until progressive disease or death (otherwise censored). The Heidelberg score was determined as described elsewhere [7]. A two-tailed p-value ≤ 0.05 was considered significant.

## Results

### Patient characteristics

Using the department’s database, 88 patients with recurrent HGG treated at our department from 5/2004 to 9/2013 were identified and retrospectively analyzed. All patient characteristics are shown in Table 1.

**Table 1:** Patient characteristics, N = 88

**Characteristic**	**Patients**
Sex	
• Male	57 (64.8%)
• Female	31 (35.2%)
Median Age [y]	51.0 (18 – 73)
• < 50	39 (44.3%)
• ≥ 50	49 (55.7%)
Median KPS	80 (40 – 100)
• KPS < 70	18 (20.5%)
• KPS ≥ 70	65 (73.9%)
• Unknown	5 (5.7%)
Median dose of primary radiotherapy	60 Gy
Median dose of re-irradiation	36 Gy
Time interval ≤ 12 months	29 (33%)
Time interval > 12 months	59 (67%)
Bevacizumab during re-irradiation	
• Yes	71 (80.7%)
• No	17 (19.3%)
MGMT methylation status	
• Methylated	42 (47.7%)
• not methylated	36 (40.9%)
• unknown	10 (11.4%)
Initial WHO grade	
• II	7 (8.0%)
• III	20 (22.7%)
• IV	61 (69.3%)
WHO grade at relapse	
• III	23 (26.1%)
• IV	65 (73.9%)
Concomitant TMZ treatment during first RT	
• Yes	68 (77.3%)
• No	20 (22.7%)
Chemotherapy	
• No adjuvant chemotherapy	36 (40.9%)
• Adjuvant therapy	45 (51.1%)
• Unknown	7 (8.0%)

8.0% of patients had a WHO grade II glioma at initial diagnosis, progressing to a secondary HGG at relapse, median age was 51 years (range, 18–73 years, 44.3% <50 years) and median KPS was 80 (range, 40–100). 77.3% of patients were treated with TMZ during adjuvant/primary RT.

Because MGMT promoter methylation status was not systematically analyzed before 2006, it is only available in 78 out of 88 patients; retrospective evaluation of MGMT-promoter methylation status was not a focus of the present manuscript.

Seventy-one patients received bevacizumab in addition to re-irradiation, 17 patients were re-irradiated without bevacizumab. Median follow-up for all patients from the start of re-irradiation was 30 months (95% CI, 12.6-47.3 months) and in 33% of all cases the interval between the end of primary irradiation and re-irradiation was ≤ 12 months.

### Survival data

Considering the course after re-irradiation, median post-recurrence progression-free survival (PR-PFS) was 4 months (95% CI, 3–5 months) and median PRS 7 months, (95% CI, 5 – 8 months) for the entire patient population.

Re-irradiation with bevacizumab was generally well tolerated (three grade 2 toxicities (3%), one grade 3 (1%), two grade 4 toxicities (2%) and one grade 5 toxicity (1%)).

When comparing both therapeutic subgroups (bevacizumab vs. no bevacizumab during re-irradiation), no statistically significant differences could be observed concerning WHO grade, age category, sex, KPS or adjuvant chemotherapy – so no bias was present towards one of the subgroups.

**Figure 1:**
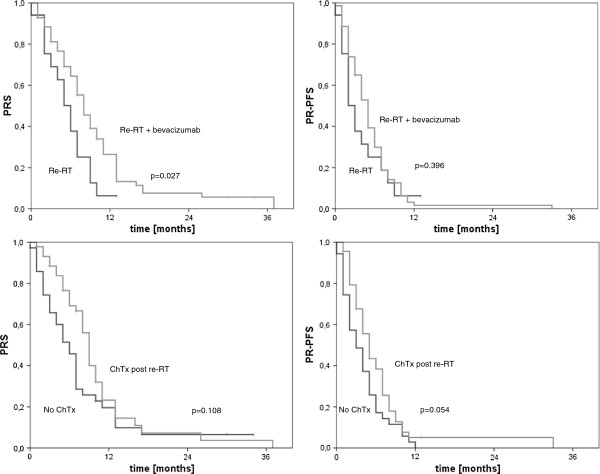
Kaplan-Meier curves for subgroups stratified by chemotherapy and application of concomitant bevacizumab, according to PRS and PR-PFS.

The results of this analysis show an association between increased PRS and PR-PFS rates and the combined treatment of re-irradiation and bevacizumab.Median PR-PFS was 3 months in the group treated with radiotherapy alone compared to 5 months with re-irradiation plus bevacizumab (p = 0.396). PFS-6 was 29.9% for re-irradiation and bevacizumab compared to re-irradiation alone with 25.1% (Figure 1). Median PRS after re-irradiation alone was 6 months, whereas median PRS after re-irradiation with additional bevacizumab increased to 8 months. This result was statistically significant (p = 0.027, Figure 1).

### Univariate analysis & prognostic score

In order to define prognostic and/or predictive factors for PRS and PR-PFS univariate testing was performed and results are shown in Table 2.

**Table 2:** Univariate analysis (log-rank test/Cox regression), influence on post-recurrence survival (PRS) and post-recurrence progression-free survival (PR-PFS)

**Variable**	**Univariate p-value PRS/PR-PFS**
**Age (< 50 y, ≥ 50 y)**	ns (p = 0.717)/ns (p = 0.854)
**KPS (< 70, ≥ 70)**	ns (p = 0.156)/ns (p = 0.095)
**MGMT (meth/not meth)**	ns (p = 0.897)/ns (p = 0.711)
**Initial WHO grade (II/III/IV)**	ns (p = 0.996)/ns (p = 0.922)
**Bevacizumab (no/yes)**	p = 0.027/ns (p = 0.396)
**Adjuvant/Salvage chemotherapy (no/yes)**	ns (p = 0.108)/ns (p = 0.054)
**Sex (male/female)**	ns (p = 0.410)/ns (p = 0.304)
**Time interval (≤ 12 y, > 12 y)**	ns (p = 0.672)/ns (p = 0.349)

N = 88, ns – not significant, meth – MGMT methylated.

Age, KPS, MGMT methylation status, initial WHO grade, sex and the time interval between the end of percutaneous primary irradiation and re-irradiation were found to be non-significant variables within the univariate analysis for both PRS and PR-PFS (p-values see Table 2).

Bevacizumab was the only variable with statistically significant impact on survival according to univariate testing (p = 0.027). Concerning PR-PFS, no significant impact of bevacizumab could be derived (p = 0.396). Another factor with a trend towards improved PR-PFS was adjuvant/salvage chemotherapy (p = 0.054), for PRS this result was less pronounced (p = 0.108), see Table 2 and Figure 1. Median PRS was 9 (with) vs. 6 months (without chemotherapy), median PR-PFS was 5 (with) vs. 3 months.

For the Heidelberg score, there was no significant influence on either PRS or PR-PFS (p = 0.664, see Table 3). As shown, the survival is relatively homogeneous among the different subgroups (PRS: median 7 (excellent) vs. 7 (good) vs. 9 (moderate) vs. 7 (poor) months). According to the subgroups stratified by bevacizumab a similar result is observed, whereas the case number for patients without bevacizumab is quite small and therefore categories such as “excellent” and “moderate” are missing. If the score values are considered, again no significant results can be observed.

**Table 3:** Outcome data concerning PRS stratified by the Heidelberg score; subgroups with and without bevacizumab are shown

**Heidelberg score/group**	**Entire cohort, PRS [months]**	**Bevacizumab, PRS [months]**	**No bevacizumab, PRS [months]**
Excellent	7	7	--
Good	7	8	2
Moderate	9	9	--
Poor	7	8	6
P-value	ns (p = 0.664)	ns (p = 508)	ns (p = 0.316)

A “poor” score consists of patients with score values of 3 or 4.

## Discussion

For certain subgroups of recurrent high-grade glioma patients re-irradiation may be a strategy to prolong survival with acceptable toxicity. The aim of this study was to analyze whether the score derived by the Heidelberg group [7] could be validated in our own mono-institutional patient cohort. We failed to validate the Heidelberg score in our mono-institutional patient cohort. Several reasons could be responsible for this finding.

One specific difference between both groups represents the application of bevacizumab in the majority of cases. In this regard, the outcome of our patient cohort compares nicely with data from other groups presented by Gutin and colleagues [17] or those of Hundsberger et al. [23]. Furthermore, the survival rate of the combined treatment is promising and PFS-6 compares favorably with data found in the literature mostly ranging from 30-50% [3,4,24]. The combined treatment approach was relatively well tolerated. Overall toxicity in our study was not higher than in the use of bevacizumab alone or in combination with other agents in patients with HGG [10,25].

As shown, the stratification by bevacizumab failed to detect subgroups where the score had prognostic meaning.

Another difference compared to the score derived by Combs et al. was the inclusion of larger tumors (up to 5 – 6 cm diameter) and multifocal disease but as shown before there was no prognostic value for larger tumors [26].

Our patient cohort seems to be very heterogeneous with a potential timing bias as some patients with initially low-grade tumors have been multimodally treated many years before re-irradiation – due to the introduction of bevacizumab and initial positive results this option became more frequently used so that results could be obtained for a more realistic patient cohort without selection bias. This explains why the historical group of patients who have only been re-irradiated is comparatively small.

Concerning heterogeneity, further aspects have to be mentioned - our cohort is substantially different to the initial cohort examined by the Heidelberg group concerning previous and maintenance therapies - namely the use of brachytherapy, re-surgery and certain chemotherapy combinations, which makes it even more difficult to derive a prognostic meaning from the time interval between both RT sessions.

Similarly to our findings, Scholtyssek and colleagues also failed to validate the Heidelberg score in their dataset [27]. Their cohort included 64 patients, no initial WHO grade II patients were present and the time interval between primary and re-irradiation had no significant impact on outcome.

Altogether, summarizing both these studies, even within the univariate analysis the factors included in the Heidelberg score were not (this work) or just in part (Scholtyssek et al.) statistically significant; therefore, the inability of validating the Heidelberg score is most likely due to the heterogeneity of the different treatment cohorts.

In conclusion, further studies and consortial data collections such as the Radplanbio database are needed to find prognostic markers in order to identify those patients who would profit most from re-irradiation and to allow for a final judgment of the Heidelberg score [28-30].

### Consent

This retrospective study was exempt from requiring ethics approval. Bavarian state law (Bayrisches Krankenhausgesetz/Bavarian Hospital Law §27 Absatz 4 Datenschutz (Dataprotection)) allows the use of patient data for research, provided that any person’s related data are kept anonymous. German radiation protection laws request a regular analysis of outcomes in the sense of quality control and assurance, thus in the case of purely retrospective studies no additional ethical approval is needed under German law.

## Competing interests

The authors declare that conflicts of interest do not exist.

## Authors’ contributions

MN & SEC planned, coordinated and performed the study. MF collected and MN analyzed the data. MN, UG, SEC & CB prepared the manuscript. All authors read and approved the final manuscript.

## Authors’ information

All authors except SEC are members of the CCC Neuro-Oncology, University of Munich.
